# Levonorgestrel emergency contraception and bodyweight: are current recommendations consistent with historic data?

**DOI:** 10.1080/21556660.2020.1725524

**Published:** 2020-02-24

**Authors:** László Kardos

**Affiliations:** Department of Infectology, University of Debrecen, Debrecen, Hungary

**Keywords:** Levonorgestrel, contraception, postcoital, treatment failure, body weight, body mass index

## Abstract

**Objective:**

To assess the consistency between current recommendations that women of body weight (BW) or body mass index (BMI) above a defined threshold should use a double dose of levonorgestrel (LNG) for emergency contraception (EC) and observed frequency of pregnancy in historic studies of single-dose LNG for EC.

**Methods:**

We applied double dose recommendation criteria to individual participant level data from three historic studies of the WHO’s Human Reproductive Program to categorize subjects into single dose-recommended (SDR) and double dose-recommended (DDR) groups and compared the latter to the former using pregnancy risk ratios (RR).

**Results:**

A total of 5859 subjects with 59 pregnancies made up the full dataset. Depending on the recommendation source (USA or UK) and inclusion or exclusion of heavy outlier data, DDR criteria were satisfied by 3.7% to 18.9% of subjects. Pregnancy proportions were mostly lower in DDR than in SDR subjects, with risk ratio estimates ranging from zero to 1.17, exceeding unity only when the USA criterion was used with outliers included. DDR subjects had a significantly lower relative frequency of pregnancy than SDR subjects when the UK criteria were used and outliers excluded (RR = 0.17 [95% CI: 0.04; 0.70], *p* = .0024).

**Conclusions:**

Our findings are consistent with the notion that there is no real loss of pregnancy control with single-dose LNG-EC in high-BMI and/or high-BW users, and today’s double dose recommendations were prematurely issued and remain questionable.

## Introduction

Levonorgestrel (LNG), an orally administered synthetic progestogen developed in the 1970s, is the active substance of a widely available and established method of emergency contraception (EC). Recently, a concern emerged that there might be a loss of efficacy (LoE) of LNG-EC associated with high body mass index (BMI) and/or bodyweight (BW).

The first indication came from a meta-analysis by Glasier et al.^1^ of two randomized controlled trials comparing the efficacy of ulipristal acetate (UPA) with that of LNG in participants from the UK, USA and Ireland^2^^,^^3^. Taking a pooled group of normal and underweight LNG-EC users as reference, the study estimated a nonsignificant doubling and a significant 4.4-fold increase in the odds of pregnancy in overweight and obese women, respectively. The findings were supported by a pooled analysis of the same two studies using various analytical approaches^4^.

At the request of the Swedish authority, a European Medicines Agency (EMA) review of LNG and UPA EC efficacy was initiated in January 2014. In addition to the meta-analysis by Glasier et al., the Agency considered a pooled analysis of the LNG arms of three EC studies by the World Health Organization (WHO) involving 17 countries in Africa, Asia, Australia, Europe and Latin America. Published later by Gemzell-Danielsson et al.^5^, the pooled analysis concluded that there was no evidence for pregnancy rates varying across BMI or BW. Importantly, this analysis identified a special subgroup of Nigerian women who were exceptionally short for their weight. When these severe outliers (very small minority of observations with highly untypical values) were included, the analysis detected a significant LoE associated with high BMI, which disappeared when they were excluded. The EMA concluded that the data available did not support the conclusion that the contraceptive effect of EC pills was reduced in women with high bodyweight/BMI and that EC could continue to be used after unprotected intercourse or contraceptive failure, as soon as possible, regardless of the woman’s bodyweight^6^.

In 2016, Jatlaoui et al.^7^ published a systematic review of secondary analyses including the meta- and pooled analyses mentioned above. The authors weighed down the strength of evidence of Gemzell-Danielsson et al.’s relative to Glasier/Kapp et al.’s assessment (5812 and 1731 LNG user subjects, respectively) because they misunderstood and misrepresented the analytic approach of the former (they criticized how BMI and BW had been split into too many categories, when in fact both were continuous variables) and concluded that women with obesity face a higher risk of pregnancy after LNG-EC than those who are normal/underweight.

A year later, a pooled analysis by Festin et al.^8^ of the three Gemzell-Danielsson studies plus a similar 1993 Hong Kong study^9^ again showed that a decrease in the contraceptive effect of LNG among obese women was technically detectable and duly pointed out that this was fully dependent on the inclusion of the Nigerian outliers.

Other sources of relevant information for judgment on the LoE issue include pharmacokinetics (PK) studies on the impact of obesity on PK parameters of EC preparations^10–13^ or regularly taken oral contraceptives containing LNG^14–16^. Generally, they found reduced systemic exposure parameters for total LNG in obese women. In the PK studies conducted by Edelman et al.^10^ and Natavio et al.^11^, parameter differences across weight categories were, however, much smaller for free LNG, the physiologically active fraction. Importantly, the effect of PK differences on ovulation suppression (OS) in an EC relation was either not evaluated in these studies or not yet reported about (2019 study by Natavio et al.).

The fact that they were not designed to determine the impact of BMI on contraceptive efficacy is an overarching limitation of all six original trials comprising the Glasier/Kapp and Gemzell-Danielsson meta-analyses. These studies had poor sample coverage of high BW/BMI individuals because they did not specifically recruit participants with any need of such coverage in mind. Also, pregnancy was generally a very rare outcome, which impacted negatively on the precision of BMI/BW effect estimates.

Even though the body of evidence available is far from conclusive, the findings above prompted two prominent professional organizations to start recommending a double dose when it comes to LNG use for EC in women with high BMI or bodyweight.

One such recommendation was formulated by The Faculty of Sexual and Reproductive Healthcare (FSRH; United Kingdom). In their 2017 EC Guideline^17^, they propose that in LNG-EC users, a double dose of the normal 1.5 mg formulation be considered for women weighing >70 kg or having a BMI >26 kg/m^2^.

A similar approach is promoted by the American Society for Emergency Contraception (ASEC) and states that a double dose may improve efficacy for women with BMI >30kg/m^2^.^18^ Both these recommendations pertain to cases where LNG is opted for after considering other choices including the copper intrauterine device and UPA.

The objective of this work was to assess the consistency between these double dose recommendations and observed frequency of pregnancy in historic studies of single dose LNG carried out many years before today’s double dose recommendations first emerged. Our working hypothesis was that if LNG users of high BW/BMI had poor pregnancy control because they took the single rather than the double dose, this should manifest in the form of elevated pregnancy rates relative to their fellow study participants who did not meet today’s double dose criteria.

## Materials and methods

We had access to individual participant level data from three historic studies of the WHO’s Human Reproductive Program^19–21^. Details of these studies as well as data handling, filtering, and outlier identification criteria are provided in our earlier publication based on the same dataset^5^. In short, all subjects in the LNG arms of these studies used a total dose of 1.5 mg (equivalent in quantity to the standard single dose). Observations with treatment delay missing, negative, or exceeding 72 h were deleted as LNG-EC is recommended to be taken within 72 h from unprotected intercourse. Descriptions and analyses were based on the per-protocol set and were carried out first including and then excluding an outlier subgroup of 60 Nigerian subjects who were exceptionally short (<145 cm) for their weight (1 underweight, 3 normal weight, 15 overweight, 41 obese), and found almost exclusively at 3 of 9 study sites in that country. These are the same subjects identified in the study by Gemzell-Danielsson^5^ and mentioned in the Festin analyses as driving regression estimates toward a false association between high BMI/BW and pregnancy. They represent 1% of the sample but 6.8% (4 of 59) of pregnancies, with a 6.7% pregnancy rate compared to 0.9% in the rest of the sample.

Subjects were categorized into “single dose recommended” (SDR) and “double dose recommended” (DDR) groups, separately by the two sets of double dose recommendation criteria (FSRH, ASEC) described above.

Non-pregnant and pregnant subjects’ data were visualized on scatter plots of BMI versus BW, with separation according to DDR criteria indicated. DDR and SDR groups were described using empirical relative frequencies of pregnancy and compared using pregnancy risk ratios (RR) with 95% confidence intervals (CI). *P* values less than 0.05 (Fisher’s exact test) were considered to indicate a significant difference; 95% CI of RR fully contained within the range 1/1.25 to 1.25 was considered to indicate equivalence. The statistical package Stata^22^ was used for data handling and analysis.

## Results

A total of 5859 subjects with 59 pregnancies made up the full dataset. After outlier exclusion, the subject count was 5799, with 55 remaining pregnancies. FSRH criteria identified 18.9% (18.2% after outlier exclusion) of the sample as potentially benefiting from a double dose, while the BMI-based ASEC criterion was satisfied by a much lower 4.4% of all subjects and 3.7% of non-outliers.

**Figure 1.**
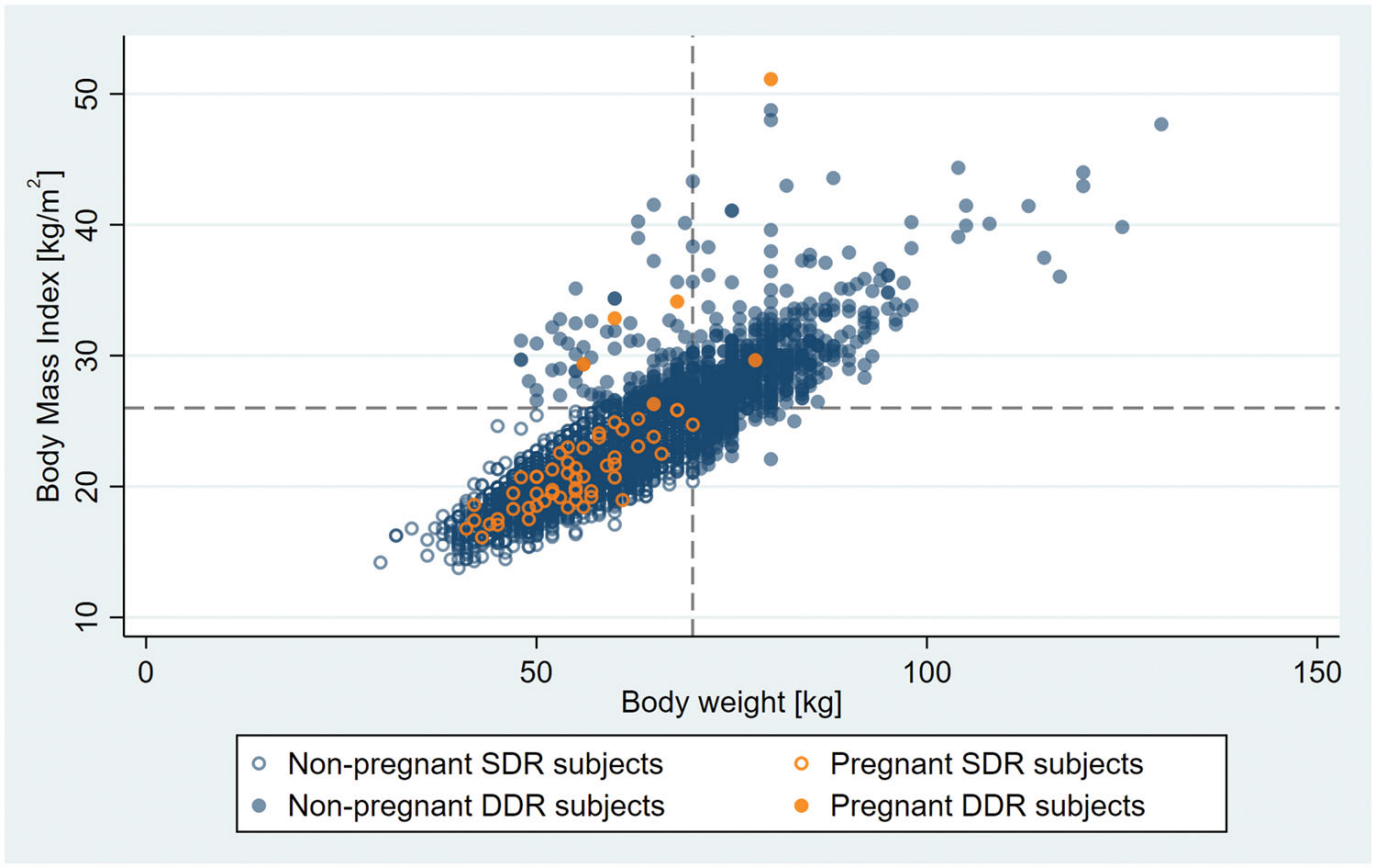
Body mass index (BMI) versus bodyweight (BW) in non-pregnant and pregnant SDR and DDR subjects. Dashed lines indicate FSRH double dose recommendation cut points on the BMI and BW scales. SDR, single dose recommended; DDR, double dose recommended.

**Figure 2.**
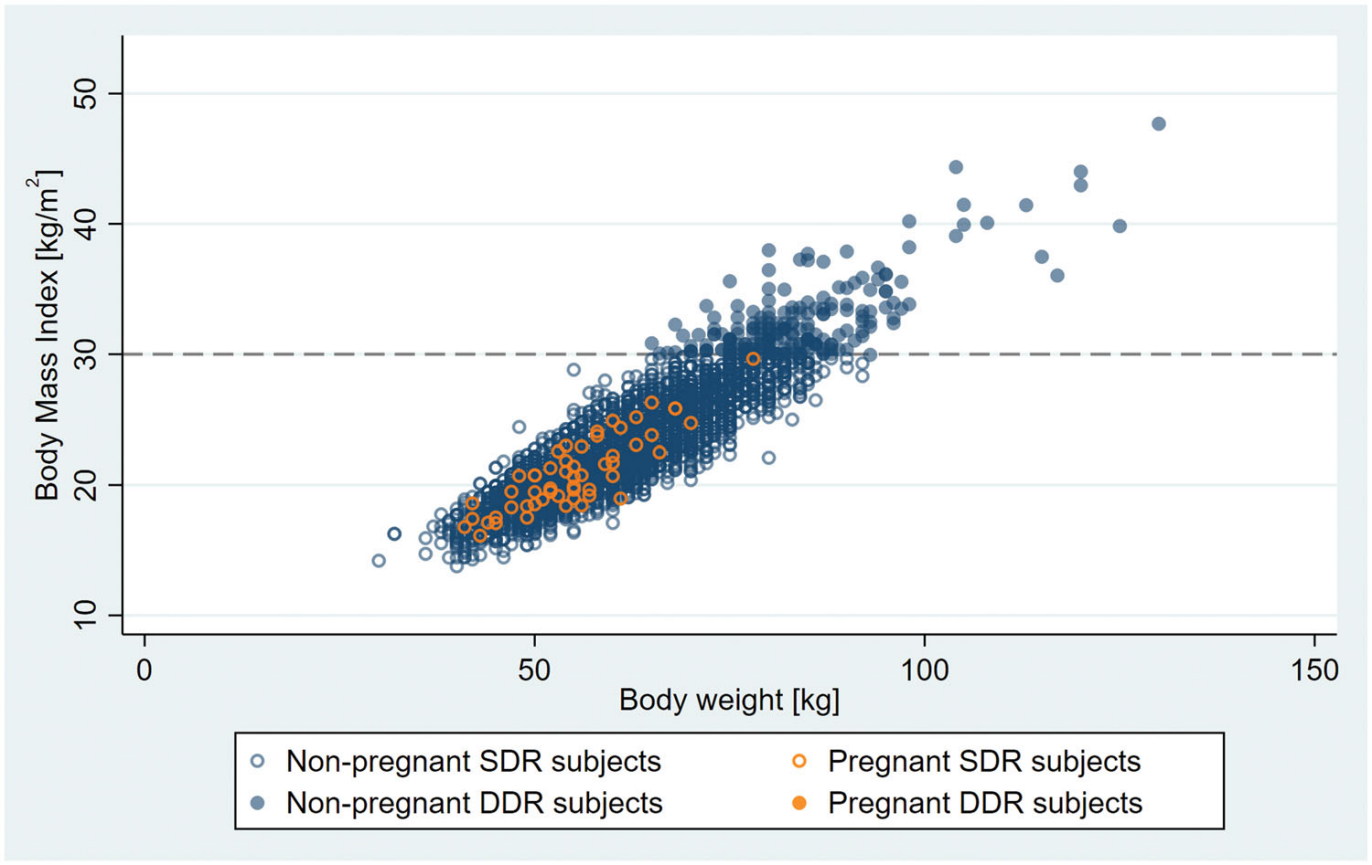
Body mass index (BMI) versus bodyweight (BW) in non-pregnant and pregnant SDR and DDR subjects with outlier observations excluded. Dashed lines indicate FSRH double dose recommendation cut points on the BMI and BW scales. SDR, single dose recommended; DDR, double dose recommended.

**Figure 3.**
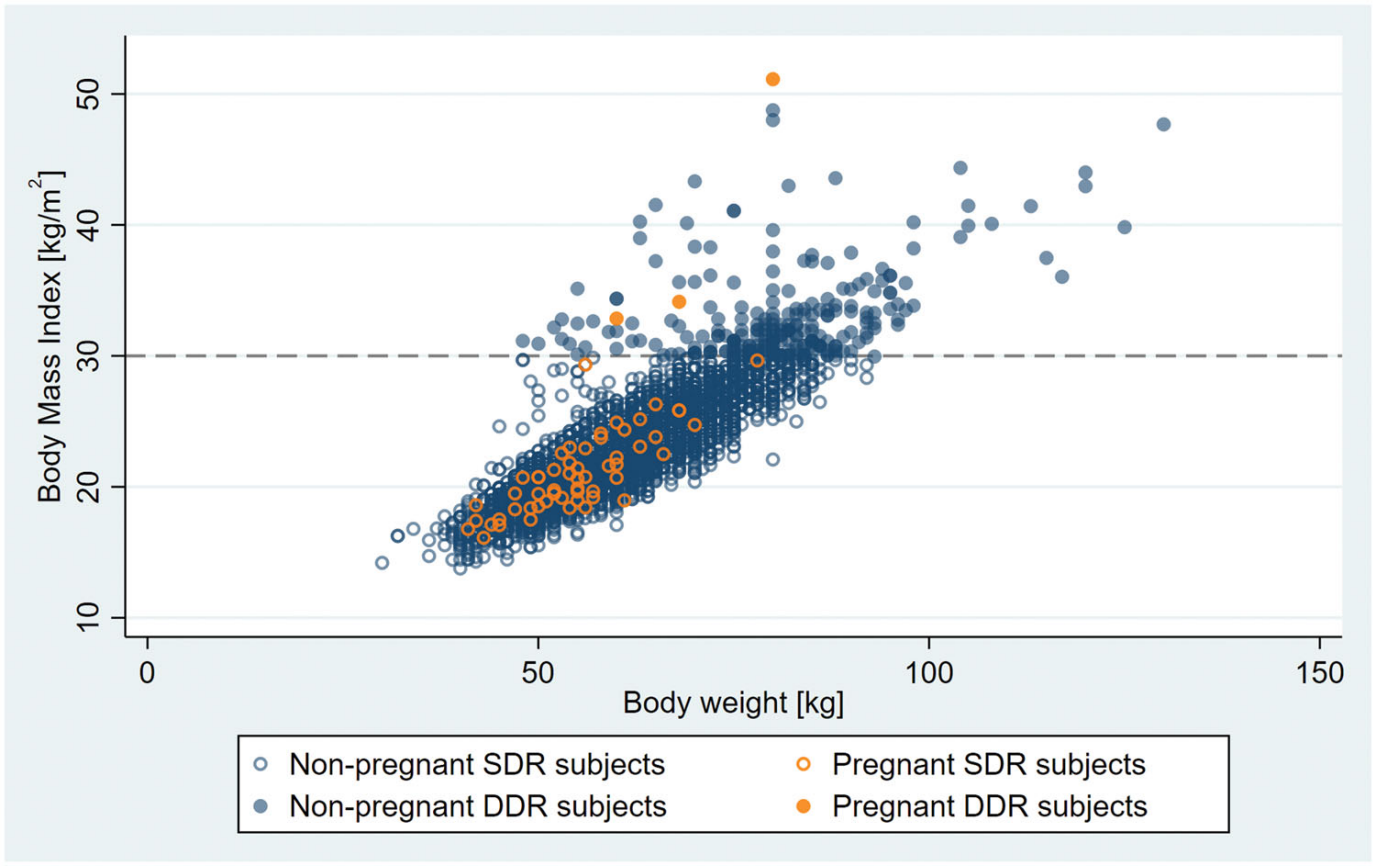
Body mass index (BMI) versus bodyweight (BW) in non-pregnant and pregnant SDR and DDR subjects. Dashed line indicates ASEC double dose recommendation cut point on the BMI scale. SDR, single dose recommended; DDR, double dose recommended.

**Figure 4.**
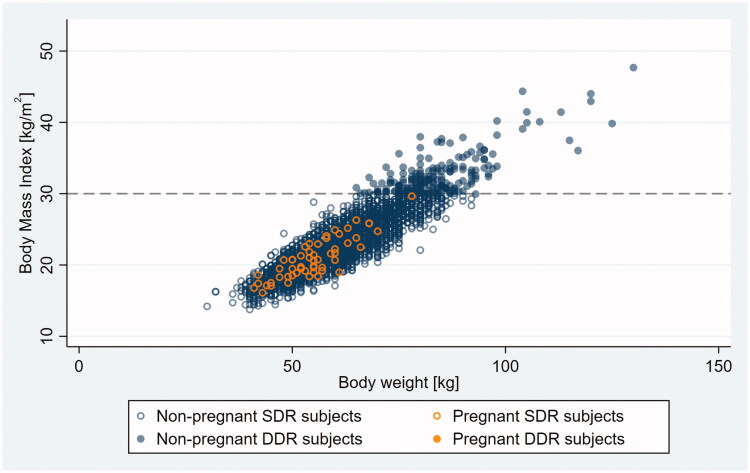
Body mass index (BMI) versus bodyweight (BW) in non-pregnant and pregnant SDR and DDR subjects with outlier observations excluded. Dashed line indicates ASEC double dose recommendation cut point on the BMI scale. SDR, single dose recommended; DDR, double dose recommended.

Outliers appeared on the scatter plot as a patch of loosely scattered observations deviating from the main body of data – a densely packed, oval-shaped cloud consistent with a linear relationship between BMI and BW – toward the higher BMI range (compare Figures 1 and 3 with Figures 2 and 4).

Pregnancy proportions in the study subsamples defined by DDR criteria ranged from 0% to 1.17% depending on outlier inclusion, while risk ratio estimates did so from zero to 1.17 (Table 1). Even though all subjects were single dosed, proportions were mostly lower in DDR than in SDR subjects (RR being below 1), rather than the other way around as expectable if tendencies in the data were consistent with the double dose approach. The only exception to this was the categorization by the ASEC criterion with outliers included. Due primarily to the sizable width of confidence intervals around rate ratio estimates, observed pregnancy rates in SDR and DDR subjects were not similar enough to satisfy predefined criteria for their equivalence in any of the comparative relations. Characterized by a risk ratio of around one-sixth, DDR subjects had a significantly lower relative frequency of pregnancy than SDR subjects when the FSRH criteria were used and outliers were excluded. No pregnancies were observed in the DDR group after the exclusion of outliers in the comparison based on the ASEC criterion.

**Table 1.** Pregnancy proportions (%) and risk ratios (RR) for SDR (single dose recommended) and DDR (double dose recommended) subjects in subsamples defined by DDR criteria and outlier inclusion.

DDR criteria by	Outliers	SDR subjects	DDR subjects	RR [95% CI]	*p* Value
FSRH	included	53/4751 = 1.12%	6/1108 = 0.54%	0.49 [0.21; 1.13]	.0947
FSRH	excluded	53/4746 = 1.12%	2/1053 = 0.19%	0.17 [0.04; 0.70]	.0024
ASEC	included	56/5603 = 1.00%	3/256 = 1.17%	1.17 [0.37; 3.72]	.7432
ASEC	excluded	55/5584 = 0.98%	0/215 = 0.00%	0.00 [ N/A ]	.2697

## Discussion

This evaluation applied currently circulated double dose recommendation criteria to historic data of single dose users from three LNG EC studies between 1998 and 2010.

If the single dose was insufficient for adequate pregnancy control in past overweight or obese users who otherwise satisfy today’s double dose recommendation criteria, it was expected to be revealed by an observable elevated frequency of pregnancy in their subgroup as compared to other users. In contrast, our data indicate generally lower pregnancy proportions in DDR than in SDR subjects.

Although equivalence was not statistically confirmed due to estimates based on very low pregnancy counts producing wide confidence intervals, subjects satisfying the then-nonexistent double dose criterion and taking a single dose experienced similar or significantly better control of pregnancy than those who would continue to be offered a single dose today. This high level of pregnancy control seen in DDR subjects might in part be explained by the fact that fecundability rates are known to significantly decrease with increasing body weight. Unfortunately, it is not possible to establish a baseline (off-EC) pregnancy rate specifically in these historic subjects who, by all three source trials’ eligibility criteria, were healthy and with regular menstrual cycles, and thus do not generally represent the overall female population in any weight category. Due to this major limitation, we cannot reliably separate the observed sufficient pregnancy control into its key components, i.e. the contraceptive efficacy of a single-dose LNG EC pill in DDR subjects and the general limiting effect of increased body weight on fecundability.

Of note, the two different DDR definitions offered by the FSRH and ASEC have an about fourfold difference between them in the percentage of users they identify as potentially benefiting from elevated dosage. While this difference might reduce if the European and US recommendations were applied to their respective local populations only, it remains doubtful whether differences in local distributions of body composition fully explain the apparent contrast between the two organizations’ views.

Unfortunately, we are not in the position to conduct a similar consistency evaluation of data from the two studies that first generated the LoE signal. These are proprietary studies with no Individual Participant Data sharing statements provided for them. Whether the LoE issue has real substance to it has long been undecided, and a straightforward, low-cost way of gaining more insight would be to carry out an overall pooled analysis of the six historic studies. However, doing so would require that the data from all these studies be made accessible to the same analyst.

A different, more resource intensive approach for future clarification efforts would be based on targeted research into both the ovarian suppression efficacy and the PK behavior of LNG as a function of BMI and/or BW within the same trial. Through appropriate design and targeted recruitment, a mixed pharmacodynamic (PD) and PK study could ensure avoiding the shortcomings of historic studies related to poor sample coverage at distribution extremes, lack of endpoints with direct clinical importance, and prohibitive rarity of outcome events.

## Conclusions

Our findings are consistent with the notion that there is no real loss of pregnancy control with single-dose LNG-EC in high-BMI and/or high-BW users, and today’s double dose recommendations were prematurely issued and remain questionable.

## Transparency

### Declaration of financial/other relationships

The author is a contract medical statistician with a clientele including Gedeon Richter Plc. (Budapest, Hungary). JDA peer reviewers on this manuscript have no relevant financial or other relationships to disclose.

## References

[1] Glasier A, Cameron ST, Blithe D, et al. Can we identify women at risk of pregnancy despite using emergency contraception? Data from randomized trials of ulipristal acetate and levonorgestrel. Contraception. 2011;84(4):363–367. 21920190 10.1016/j.contraception.2011.02.009

[2] Creinin MD, Schlaff W, Archer DF, et al. Progesterone receptor modulator for emergency contraception. Obstetrics Gynecol. 2006;108(5):1089–1097. 10.1097/01.AOG.0000239440.02284.45 PMC2853373 17077229

[3] Glasier AF, Cameron ST, Fine PM, et al. Ulipristal acetate versus levonorgestrel for emergency contraception: a randomised non-inferiority trial and meta-analysis. The Lancet. 2010;375(9714):555–562. 10.1016/S0140-6736(10)60101-8 20116841

[4] Kapp N, Abitbol JL, Mathé H, et al. Effect of body weight and BMI on the efficacy of levonorgestrel emergency contraception. Contraception. 2015;91(2):97–104. 25528415 10.1016/j.contraception.2014.11.001

[5] Gemzell-Danielsson K, Kardos L, von Hertzen H. Impact of bodyweight/body mass index on the effectiveness of emergency contraception with levonorgestrel: a pooled-analysis of three randomized controlled trials. Curr Med Res Opin. 2015;31(12):2241–2248. 26368848 10.1185/03007995.2015.1094455

[6] European Medicines Agency. Levonorgestrel and ulipristal remain suitable emergency contraceptives for all women, regardless of bodyweight. 2014. Available from: http://www.ema.europa.eu/ema/index.jsp?curl=pages/news_and_events/news/2014/07/news_detail_002145.jsp&mid=WC0b01ac058004d5c1

[7] Jatlaoui TC, Curtis KM. Safety and effectiveness data for emergency contraceptive pills among women with obesity: a systematic review. Contraception. 2016;94(6):605–611. 27234874 10.1016/j.contraception.2016.05.002 PMC6511981

[8] Festin MPR, Peregoudov A, Seuc A, et al. Effect of BMI and body weight on pregnancy rates with LNG as emergency contraception: analysis of four WHO HRP studies. Contraception. 2017;95(1):50–54. 27527670 10.1016/j.contraception.2016.08.001 PMC5357708

[9] Ho PC, Kwan M. A prospective randomized comparison of levonorgestrel with the Yuzpe regimen in post-coital contraception. Human Reprod. 1993;8(3):389–392. 1993/03 10.1093/oxfordjournals.humrep.a138057 8473453

[10] Edelman AB, Cherala G, Blue SW, et al. Impact of obesity on the pharmacokinetics of levonorgestrel-based emergency contraception: single and double dosing. Contraception. 2016;94(1):52–57. 27000996 10.1016/j.contraception.2016.03.006 PMC4944814

[11] Natavio MF, Diaz OV, Wilson ML, et al. Pharmacokinetics of the levonorgestrel-only emergency contraceptive regimen among normal-weight, obese and extremely obese users: a pilot study. Contraception. 2016;94(4):418.

[12] Praditpan P, Hamouie A, Basaraba CN, et al. Pharmacokinetics of levonorgestrel and ulipristal acetate emergency contraception in women with normal and obese body mass index. Contraception. 2017;95(5):464–469. 28126541 10.1016/j.contraception.2017.01.004

[13] Natavio M, Stanczyk FZ, Molins EAG, et al. Pharmacokinetics of the 1.5 mg levonorgestrel emergency contraceptive in women with normal, obese and extremely obese body mass index. Contraception. 2019;99(5):306–311. 30703352 10.1016/j.contraception.2019.01.003 PMC6499670

[14] Westhoff CL, Torgal AH, Mayeda ER, et al. Ovarian suppression in normal-weight and obese women during oral contraceptive use. Obstetrics Gynecol. 2010;116(2, Part 1):275–283. 10.1097/AOG.0b013e3181e79440 20664386

[15] Westhoff CL, Torgal AH, Mayeda ER, et al. Pharmacokinetics of a combined oral contraceptive in obese and normal-weight women. Contraception. 2010;81(6):474–480. 20472113 10.1016/j.contraception.2010.01.016 PMC3522459

[16] Luo D, Westhoff CL, Edelman AB, et al. Altered pharmacokinetics of combined oral contraceptives in obesity - multistudy assessment. Contraception. 2019;99(4):256–263. 30684471 10.1016/j.contraception.2018.12.009 PMC6441376

[17] Faculty of Sexual & Reproductive Healthcare (FSRH). Emergency contraception March 2017 (Updated December 2017). 2017. Available from: https://www.fsrh.org/standards-and-guidance/current-clinical-guidance/emergency-contraception/

[18] American Society for Emergency Contraception (ASEC). Statement: Efficacy of emergency contraception and body weight: current understanding and recommendations. 2016. Available from: http://www.americansocietyforec.org/uploads/3/4/5/6/34568220/asec_ec_efficacy_and_weight_statement.pdf

[19] Regulation TFoPMoF. Randomised controlled trial of levonorgestrel versus the Yuzpe regimen of combined oral contraceptives for emergency contraception. The Lancet. 1998;352(9126):428–433. 9708750

[20] von Hertzen H, Piaggio G, Peregoudov A, et al. Low dose mifepristone and two regimens of levonorgestrel for emergency contraception: a WHO multicentre randomised trial. The Lancet. 2002;360(9348):1803–1810. 10.1016/S0140-6736(02)11767-3 12480356

[21] Dada OA, Godfrey EM, Piaggio G, et al. A randomized, double-blind, noninferiority study to compare two regimens of levonorgestrel for emergency contraception in Nigeria. Contraception. 2010;82(4):373–378. 20851232 10.1016/j.contraception.2010.06.004

[22] StataCorp. Stata Statistical Software: Release 15. College Station (TX): StataCorp LLC; 2017.

